# Vision-based dirt distribution mapping using deep learning

**DOI:** 10.1038/s41598-023-38538-3

**Published:** 2023-08-06

**Authors:** Ishneet Sukhvinder Singh, I. D. Wijegunawardana, S. M. Bhagya P. Samarakoon, M. A. Viraj J. Muthugala, Mohan Rajesh Elara

**Affiliations:** 1 Engineering Product Development Pillar, Singapore University of Technology and Design, Singapore, 487372 Singapore; 2 Temasek Junior College, Singapore, 469278 Singapore

**Keywords:** Electrical and electronic engineering, Mechanical engineering

## Abstract

Cleaning is a fundamental routine task in human life that is now handed over to leading-edge technologies such as robotics and artificial intelligence. Various floor-cleaning robots have been developed with different cleaning functionalities, such as vacuuming and scrubbing. However, failures can occur when a robot tries to clean an incompatible dirt type. These situations will not only reduce the efficiency of the robot but also impose severe damage to the robots. Therefore, developing effective methods to classify the cleaning tasks performed in different regions and assign them to the respective cleaning agent has become a trending research domain. This article proposes a vision-based system that employs YOLOv5 and DeepSORT algorithms to detect and classify dirt to create a dirt distribution map that indicates the regions to be assigned for different cleaning requirements. This map would be useful for a collaborative cleaning framework for deploying each cleaning robot to its respective region to achieve an uninterrupted and energy-efficient operation. The proposed method can be executed with any mobile robot and on any surface and dirt, achieving high accuracy of 81.0%, for dirt indication in the dirt distribution map.

## Introduction

Cleaning is typically considered monotonous work mainly performed in dirty and unfavourable environments^1^. It can even be associated with dangerous objects and locations or cause cumulative trauma disorders in human labour^2^. Cleaning is an essential task for maintaining living standards. Therefore, in recent times, cleaning robots have been at the forefront as an ideal solution for this problem^3^. Developments in cleaning robots over the last 20 years have been focusing on empowering their autonomy in order to improve their performance. Many cleaning robotic devices have been developed to clean floors^4^, facades^5^, swimming pools^6^, ventilation ducts^7^, and staircases^8^. All these robots come with unique mechanisms and autonomy strategies specifically optimized for their respective cleaning tasks.

However, cleaning robots for facades, pipes, ventilation ducts, and sewer lines are not yet mass-produced. These systems are specially optimized for the requirements and geometry of the surface or object being cleaned and are used exclusively in professional environments rather than in the residential sector^3^. However, devices such as floor-cleaning robots have created mass markets with significant revenue. The global market for cleaning robots was valued at USD 8.34 billion in 2021, with a compound annual growth rate of 22.7%^9^. For instance, vacuuming robots for household use are among the most widely sold robot systems worldwide. Area coverage^10^, energy usage^11^, coverage time^12^, reliability and safety^13^, and human-comfort^14^ are the widely expected features of cleaning robots.

Vacuuming, scrubbing, and wet mopping are distinct tasks performed by floor-cleaning robots. Deploying the appropriate robot for each cleaning task can improve efficiency^15^. A framework that enables coordinated operations among various robots, such as vacuuming robots, mopping robots, and scrubbing robots, to address different cleaning requirements can be used to enhance cleaning efficiency. This sort of a framework is referred to as a collaborative cleaning framework in the scope of this paper. Moreover, a collaborative cleaning framework can avoid failures and potential damage to the robots or the environment as a result of employing incompatible cleaning robots for specific tasks. For instance, a vacuum cleaning robot attempting to absorb liquids or a wet cleaning robot encountering solid dirt can result in undesired outcomes.

In this regard, a collaborative cleaning framework with a set of heterogeneous cleaning robots should be able to identify the cleaning requirements in each location of an environment of interest. The role of an inspection robot can be introduced for a collaborative cleaning framework for realizing this necessity. Here, the inspection robot identifies the cleaning requirement for each area and guides the respective cleaning agent to identified locations while preventing unnecessary coverage to ensure efficiency and reliability. For example, a moping robot is sent to locations with liquid spills while sending the vacuum robot to locations with dust. This strategy ensures efficient energy usage and prevents possible damage to the vacuum robot due to liquid. This sort of an inspection robot can have a simple design with low energy consumption and should only be equipped with a system to inspect the environment. However, this sort of concept has yet to be fully realized, and merely a few supporting concepts can be found in the literature. For example, Ramalingam et al.^16^ proposed a closed-circuit television (CCTV)-based system to guide a robot for selective cleaning. Here, the dirt location and human activities are detected, and an optimum path will be created for the robot for spot cleaning resulting in higher efficiency. However, the system could not consider different dirt types and the use of a set of robots with different cleaning functions.

In order to succeed in this concept, the perception system of the inspection robot should be capable of classifying the dirt and creating a dirt distribution map of the environment that can be used to guide the set of cleaning robots with different cleaning functions. In recent times, perception requirements in mobile robotics have been mostly fulfilled with computer vision and artificial intelligence (AI)^17^. A multitude of research has been performed to classify the dirt types available on a floor to improve the performance of cleaning robots. Among these, developments of systems for mud and dirt separation^18^, liquid and solid dirt separation^19^, stain detection^20^, and dirt type classification of office environments^21^ could be found. However, the scope of much of the work is limited to the classification of dirt, and the creation of dirt distribution maps to be used by a set of cleaning robots with heterogeneous capabilities has not been discussed.

This article proposes a novel dirt distribution mapping method, which is specifically designed for use in a collaborative cleaning framework with a set of heterogeneous robots that have unique cleaning features. The proposed mapping method is developed using YOLOv5 and DeepSORT algorithms to detect, classify and track dirt which subsequently creates a map that indicates the regions to be assigned for different cleaning requirements. Vacuuming, mopping, and scrubbing are considered the cleaning requirements in this work. This map is expected to be used by a collaborative cleaning framework with a set of heterogeneous cleaning robots each having unique cleaning features. Thus, the work proposed in this paper would contribute to the development of the field of robot-aided floor cleaning, as mapping dirt distribution into the three categories of solid, liquid, and stain has not been reported in the literature. The methodology followed to achieve the proposed goal is presented in the “Method” Section. The “Results and discussion” Section, discusses the experimental validation of the proposed system. Conclusions of the work are given in the “Conclusion” Section.

## Method

### Overview of the proposed system

**Figure 1:**
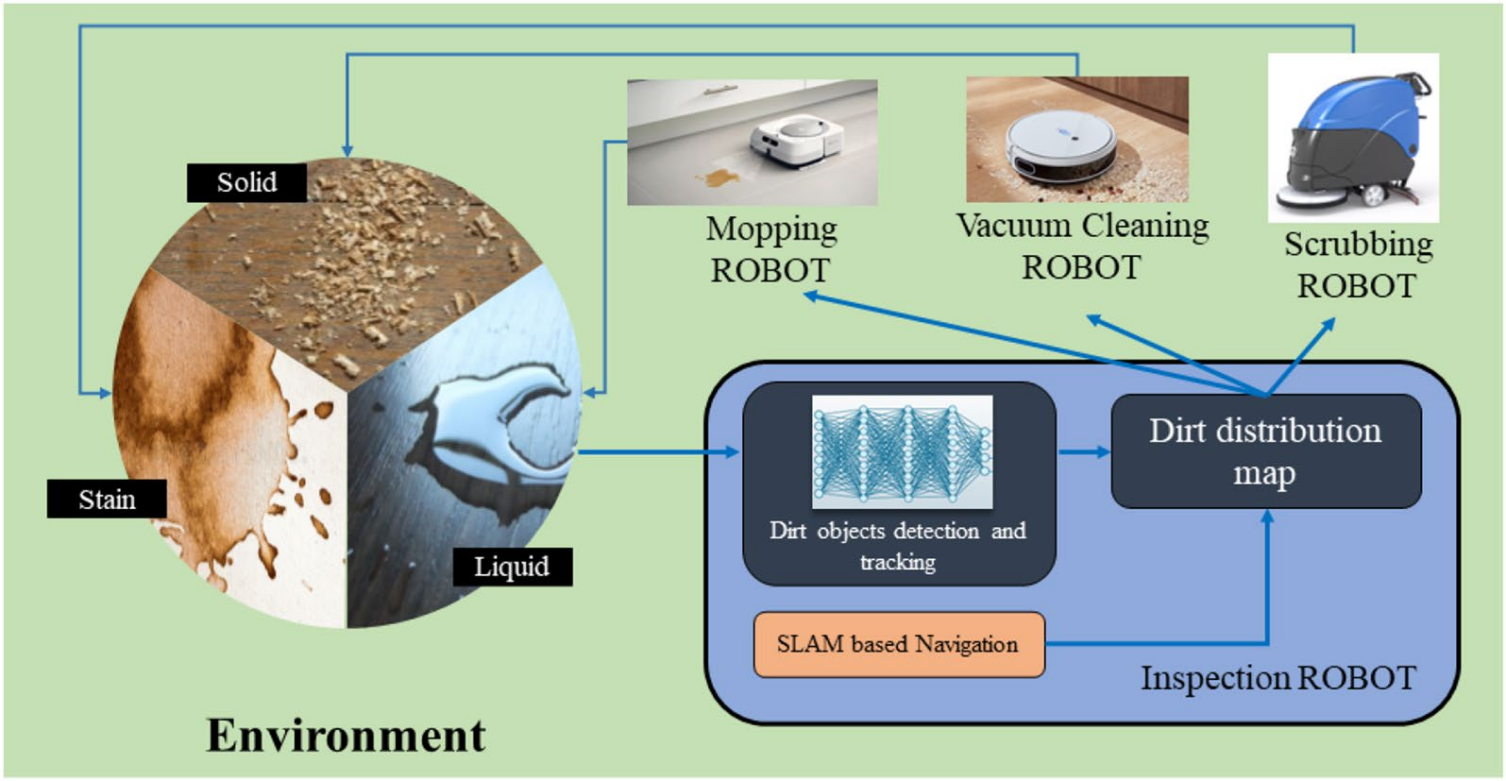
Overview of the proposed dirt mapping method in a collaborative cleaning framework with a set of heterogeneous robots. The development of the dirt mapping method for the inspection robot is addressed within the scope of this paper.

An overview of the concept of a collaborative cleaning framework with a set of heterogeneous robots that have unique cleaning functions is depicted in Fig. 1. This sort of a collaborative cleaning framework with a set of heterogeneous robots requires to know the dirt distribution in the environment along with dirt types to assigned the robots for efficient and effective cleaning. For example, the mopping robot will be assigned to the liquid dirt, the scrubbing robot will be dispatched to the stain marks, and the vacuum cleaning robot will be responsible for cleaning the solid dirt. This strategy would prevent the possible damages in events of using incompatible robots (e.g., use of a vacuum robot for a liquid spill). In this regard, a map that indicates the dirt locations in a selected area with the corresponding class (solid dirt, liquid spills and stain marks are considered the classes) should be created by an inspection robot dedicated for the map creation. Therefore, the development of the dirt mapping method (tagged with dirt classes) for the inspection robot is addressed within the scope of this work to realize the goal of a collaborative cleaning framework with a set of heterogeneous robots.

### Object detection and tracking system

The proposed system uses YOLOv5 to detect and classify dirt. YOLOv5 model is chosen for this work since YOLOv5 is a fast, accurate, and lightweight object detection model that is ideal for applications that require both object detection and tracking^22,23^. One of the strengths of deep convolutional neural network-based classifiers like YOLOv5 for object detection is their ability to learn features automatically from the input image data during training. This means that there is no need to manually provide features for the network. Furthermore, YOLOv5 has a smaller memory footprint than other models, making it suitable for deployment on resource-constrained devices such as mobile robots. A tracking method has been implemented to avoid repetitive detection of the same object while the robot is navigating. In addition, the use of a tracker can improve the detection and detection rate. Thus, DeepSORT is used for tracking where successful detections made by the YOLOv5 algorithm are fed into the DeepSORT algorithm for tracking and assigning a unique identity.

#### YOLOv5

**Figure 2:**
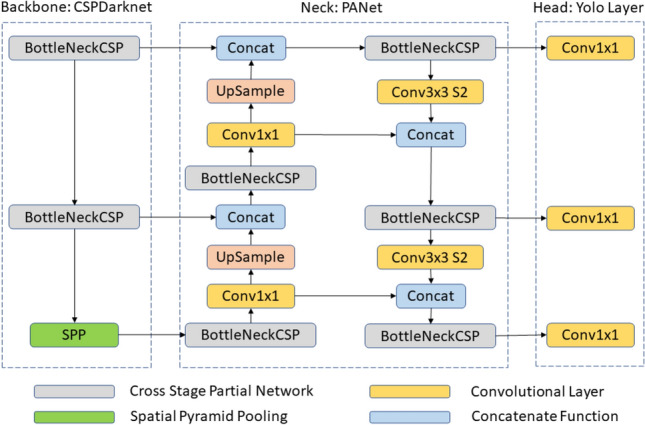
Architecture of YOLOv5.

“You Only Look Once”, also known as YOLO, is a common single-stage object detection algorithm. The YOLO algorithm is able to identify objects in a given image based on the classes defined. The algorithm outputs the bounding boxes around the detected objects, the class of each object and the detection confidence score. According to the literature, the inference speed of the YOLOv5 networks has the ability to be used in real-time object detection with good accuracy^22,23^. This ability has been confirmed by numerous detection applications such as environmental monitoring^24^ and quality control processing^25^. This high inference speed is achieved due to its unique design^26,27^. The architecture of YOLOv5 is depicted in Fig. 2. It can be divided into three segments, backbone, neck, and head. The Cross Stage Partial network (CSPnet) is the backbone of the YOLOv5 architecture and is mainly responsible for the extraction of informative features to aid in object detection. This helps reduce the spatial resolution of the image and increases its feature (channel) resolution. It uses cross-channel pooling^28^ to compress the feature maps during the feature pyramid-generating process. Hence, it is able to cut down on 75% memory usage^29^ when extracting features coupled with its low computational requirement and serves as an effective feature extractor for our dirt mapping system. The neck segment performs the feature fusion. The head section of the YOLOv5 architecture is composed of three convolution layers that predict the location of the bounding boxes, the prediction scores and the object classes. This information is then passed to the DeepSORT algorithm.

#### DeepSORT

**Figure 3:**
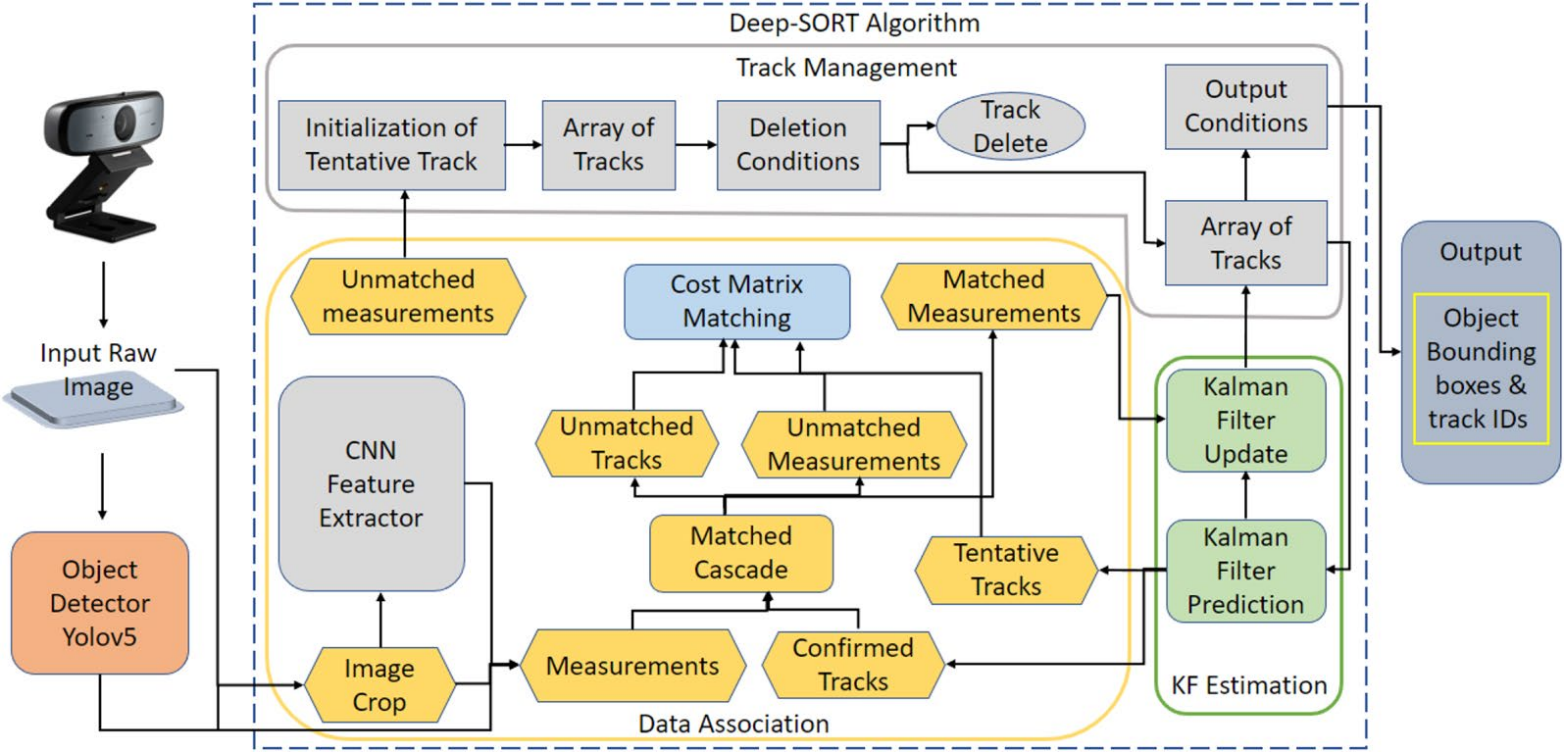
Overview of the object tracking DeepSORT algorithm.

DeepSORT is a step up from the traditional Simple Online and Real-time Tracking (SORT) algorithm^30^. It is used to track objects between two successive frames. An overview of the DeepSORT algorithm is given in Fig. 3. DeepSORT uses the Hungarian algorithm^31^ together with SORT to distinguish between detected objects in consecutive frames and is thus able to assign a unique identity to each detected object. Kalman filtering^32^ is then used to predict the future positions of the detected objects. During the track management, object tracks that meet certain conditions, such as no corresponding detection for a set number of frames, high uncertainty or low confidence for a certain duration, or exceeding a maximum track age, are deleted to ensure accurate and relevant tracks and robustness to occlusions and noise in detection data. Furthermore, with the addition of deep learning it is able to minimize identity switches as the object moves in consecutive frames. In this work, the bounding box coordinates of dirt objects detected by YOLOv5 are passed to DeepSORT with the frame for tracking. According to bounding box coordinates and object appearance, DeepSORT assigns each dirt a unique identification number and performs the tracking. The outputs of the DeepSORT algorithm is used to create the dirt distribution maps.

#### Data collection and training

For training the proposed model, we combined publicly available dirt datasets with our own new data to produce a dataset to be used. The work^33^ proved the effectiveness of using synthetic data to train a dirt detection model. Thus, we used the synthetically generated dataset provided in^33^ as a part of our dataset. We also combined the ACIN dataset^34^ as it consists of real images with severe lighting conditions, complex floor patterns and blurred images to provide a more robust dataset. Furthermore, as the ACIN dataset only consisted of a few hundred images, we also collected our own data with an RGB camera across various floor patterns, angles and lighting conditions to create a well-balanced dataset. After combining our dataset with the publicly available datasets (i.e., synthetic dataset in^33^ and ACIN dataset), we applied data augmentations techniques such as flipping and rotations to diversify our dataset further. This augmentation can reduce the CNN learning rate as well as reduces over fitting. Furthermore, the captured images were resized into 460 $\times$ 460 pixels to reduce the computing load in the training stage. This results in a labelled dataset of 2858 images with the various classes of dirt (i.e., solid, liquid, and stain). This dataset was split into training, validation, and testing in the ratio 70:20:10, respectively, for the effectiveness as suggested in^35,36^.

Transfer Learning^37^ was implemented by freezing the backbone, taking advantage of the pre-trained weights on the COCO dataset. Hence, only the last layer of the detector head is modified to detect the solid, liquid, and stains. The optimizer used was the Stochastic Gradient Descent (SGD)^38^ with a decay of 0.0005 and a learning rate of 0.01. The batch size was set to 16, and the models were trained for 50 epochs, saving the best weights. The training was done with a Nvidia GeForce GT 730 GPU and an Intel(R) Core(TM) i5-8400 CPU @ 2.80GHz 2.81 GHz CPU.

### Creating the dirt distribution map

The trained visual detection and tracking systems are deployed on a mobile robot platform to create a map that indicates the dirt distribution of the selected environment along with the dirt type. Here, the transformation of the coordinates from the camera sensor to the world frame is essential to get the global coordinates of the dirt location. Subsequently, these coordinates are transferred to the map to indicate the dirt type at the respective location.

**Figure 4:**
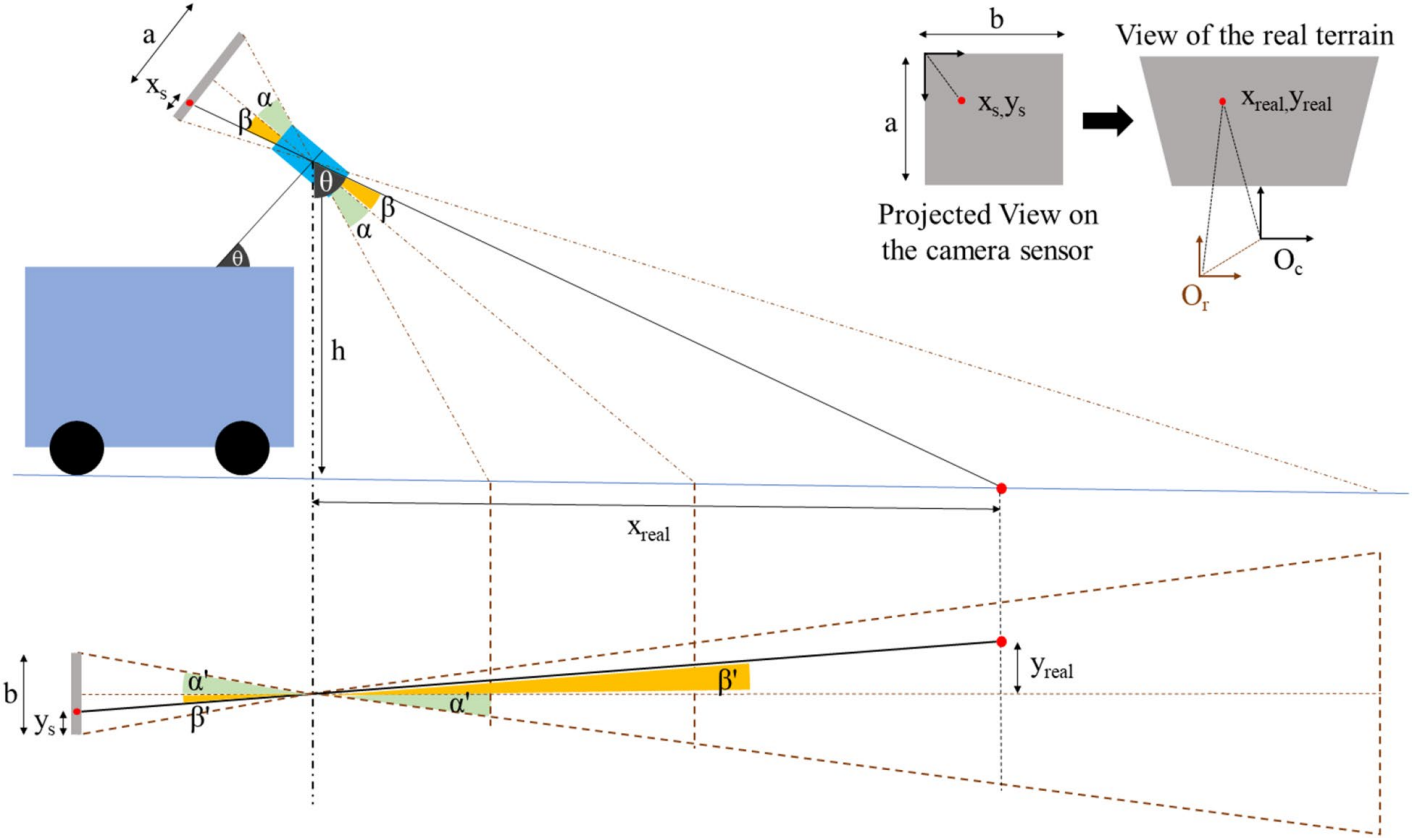
Transformation of the camera display coordinates to the world coordinate system.

This transformation can be explained with the aid of Fig. 4. The object’s projection in the camera sensor $(x_{s},y_{s})$ can be transformed to $(x_{real},y_{real})$ coordinates, which are with respect to the camera frame, using the Eqs. (1) and (2). Here, the angles $\alpha$ and $\alpha '$ represent the field of view of the camera in vertical and horizontal planes, respectively. The variables represented by $\beta$ and $\beta '$ are redundant from the equation. However, it has been indicated in the diagram for convenience. Variables *a* and *b* respectively denotes the length and width of the camera sensor in pixel while the $\theta$ represents the angle of the camera’s centre axis to the vertical line. The height to the camera from the ground is represented by *h*.

1 $$\begin{aligned}{} & {} x_{real} = h\times \tan {\left\{ \theta +\tan ^{-1}{\left[ \tan {\alpha _{1}}-\frac{x_{s}}{a} \times \left( \tan {\alpha _{1}} + \tan {\alpha _{2}} \right) \right] }\right\} }\end{aligned}$$

2 $$\begin{aligned}{} & {} y_{real} = x_{real}\times \tan {\alpha '} \times \left( 1-\frac{2y_{s}}{b} \right) \end{aligned}$$

3 $$\begin{aligned}{} & {} \vec {O_{m}P} = \vec {O_{m}O_{r}} + \vec {O_{r}O_{c}} + \vec {O_{c}P} \end{aligned}$$

After transforming the dirt coordinates with respective to the camera origin ($O_{c}$), another linear transformation has been done to obtain the coordinates with respective to the robot’s origin ($O_{r}$). Finally, using the odometry data of the robot with respective to the map origin, the dirt locations has been obtained with respective to the map origin ($O_{m}$). Equation (3) can be used to transform the point *P* in the floor from camera frame to map frame.

**Figure 5:**
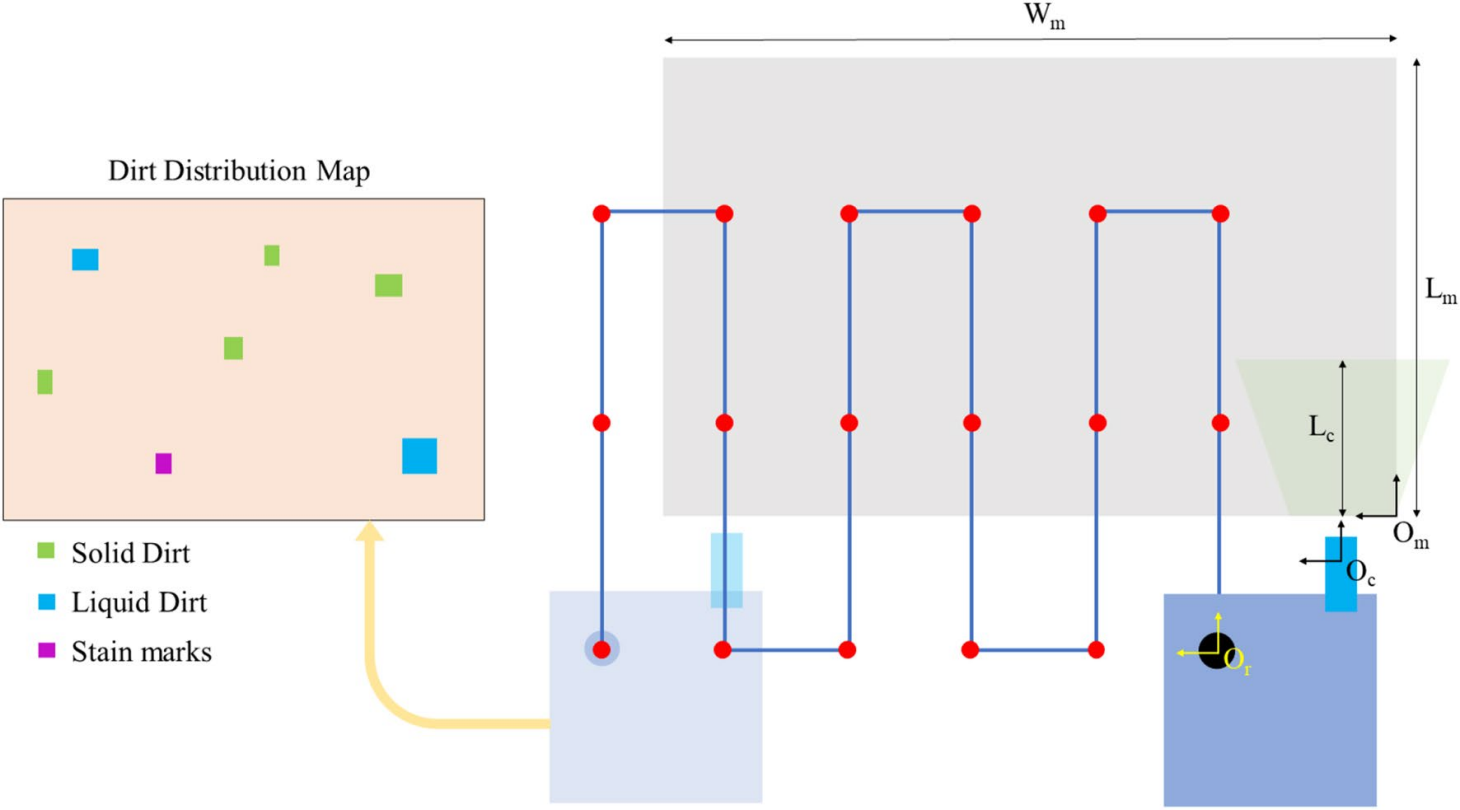
Proposed inspection strategy.

In order to create the map, the inspection robot has to be autonomously navigated in a boustrophedon motion pattern to completely inspect the selected area with $L_{m} \times W_{m}$ dimension as shown in Fig. 5. During the motion, the robot’s location and the information about the identified dirt are logged. Identification is only performed when the robot is stationary at the each way point. The distance between the way points is selected to minimize the overlapping of coverage. After the complete inspection the dirt map was created using the above mentioned coordinate transformations.

### Robot platform and the autonomy architecture

The hTetro robot in the square shape configuration without the cleaning tools was used as the inspection robot for this work. The robot measures 50 cm in total length and width with a height of 10 cm and is capable of holonomic motion using mecanum wheels powered by geared DC motors equipped with encoders.

**Figure 6:**
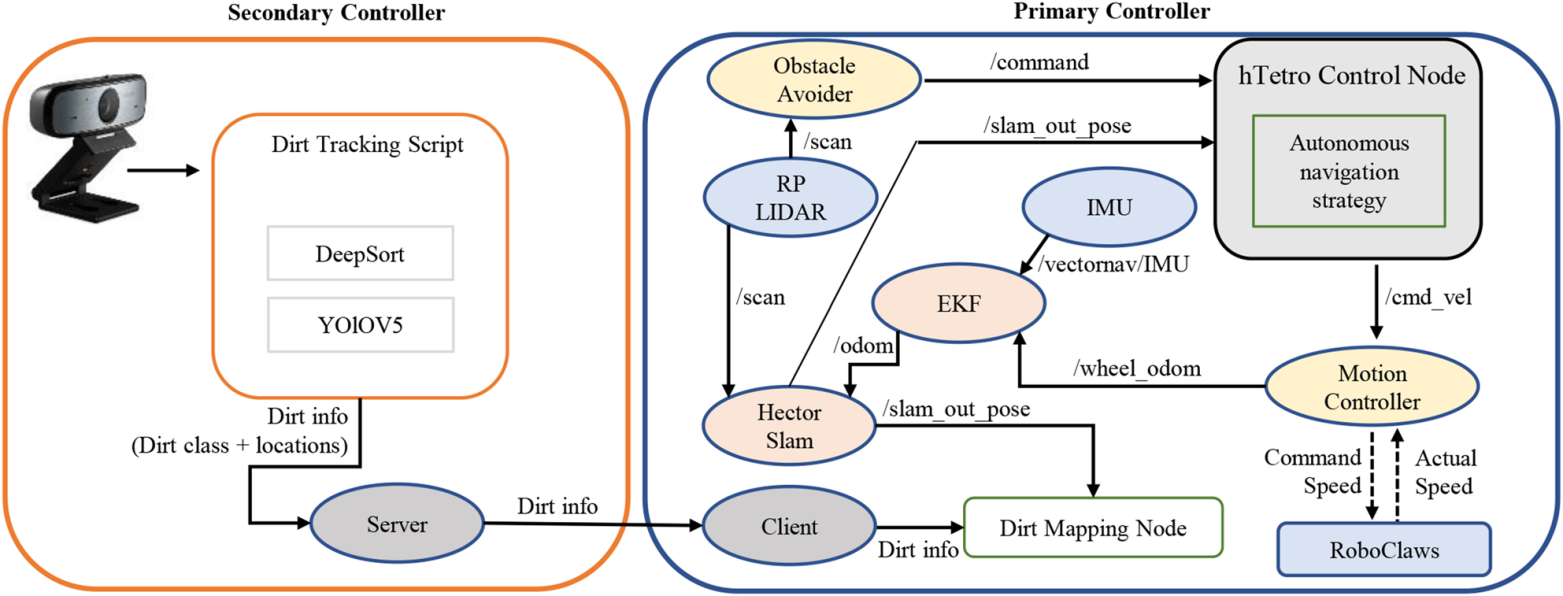
ROS-based autonomy architecture for locomotion and dirt mapping.

The robot is autonomously controlled using Robot Operating System (ROS) and was developed with two controllers: a primary controller for robot locomotion and a secondary controller for dirt tracking (See Fig. 6). The primary controller comprises three components that enable autonomous navigation in a predefined environment. This architecture involves retrieving feedback from sensors, computing the best possible path, and issuing control commands to motors to follow the path. A Raspberry Pi3 controller is used to implement the primary controller. The robot uses a predefined navigation map to create the global path, which is created using the LIDAR (RP-LIDAR A3) and the hector SLAM ROS package. The robot creates its local path using feedback from the LIDAR sensor to avoid dynamic obstacles in its environment. The wheel encoders, LIDAR sensor, and IMU sensor (Vectornav V-100) provide feedback for robot localization. The robot can be navigated by assigning waypoints, and its global path plan is defined as a boustrophedon motion pattern.

The deep learning model is executed in the secondary controller simultaneously. A laptop and a webcam are installed on the robot for this purpose. Dirt detection data, including the dirt class and bounding box coordinates with respect to the robot, are transferred to the main controller through a wireless communication protocol (python WIFI socket). A parallelly executing ROS node is used in the primary controller to subscribe to the robot position and dirt detection data. This data tagging facilitates the creation of a dirt distribution map with respect to the global coordinate system. Overall, the robot’s autonomy and the deep learning model work together to create a comprehensive dirt mapping system.

## Results and discussion

Evaluation of the effectiveness and accuracy of the proposed method has been carried out in two stages. Initially, the vision based detection and tracking system which uses the YOLOv5 and DeepSORT algorithms was evaluated based on the standard statistical measures and compared with the benchmark object detection algorithms. Subsequently, field experiments in two preset environments were performed using the htetro robot to evaluate the accuracy of the generated dirt distribution map. Deep learning models proposed in this article were developed in Tensor-flow 2.11 Win11 version and trained in a hardware configuration of Intel(R) Core(TM) i5-8400 CPU @ 2.81 GHz CPU and Nvidia GeForce GT 730 Graphics Cards. The same device was used for the testing end evaluations tasks as well.

### Evaluation of the detection and tracking model

#### Performance evaluation of the detection model

**Figure 7:**
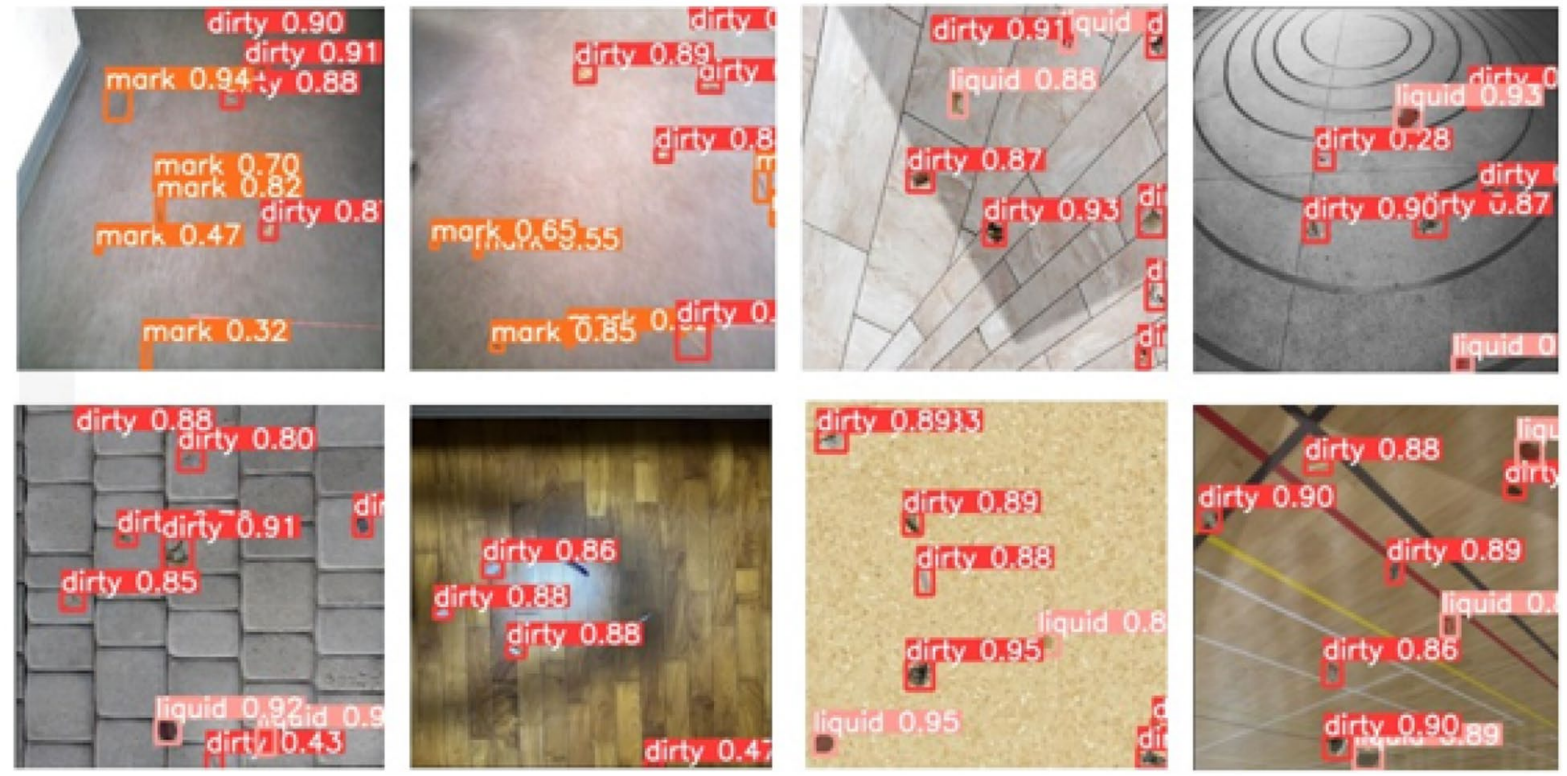
Successful detections of training images. Here, the class ‘dirty’ on the bounding boxes refers to solid dirt and the class ‘mark’ refers to stains while liquid is referred to as ‘liquid’.

The detection model successfully classified random dirt-mixed samples into three classes: solid, liquid, and stains. A set of sample results is shown in Fig. 7. In order to assess the performance of the proposed scheme, standard statistical measures, accuracy (*A*), precision (*P*), recall (*R*), $F_{measure}$, and mean average precision (*mAP*), were used. These measures could provide a comprehensive assessment of the proposed performance of the models. The calculation of these measures are given in Eqs. (4), (5), (6), (7), and (8) for *A*, *P*, *R*, $F_{measure}$, and *mAP*, respectively. *mAP* is numerically equal to the average value of the average precision (*AP*) sum across all the categories, and this measure is used to evaluate the overall performance of the model. Average precision (*AP*) is defined as the precision across all elements of a dirt category as explained in Eq. (9). Here, *tp*, *fp*, *tn*, *fn* represent the true positives, false positives, true negatives, and false negatives, respectively, as per the standard confusion matrix.

The results for overall and each dirt category detection in terms of these statistical measures are given in Table 1. The intersection of Union (*IoU*) of 0.5 was considered here. These performance matrices verified the ability of the trained YOLOv5 architecture to detect all the dirt classes with high accuracy. The performance metrics for the ‘stain’ class are relatively lower than the solid and liquid classes. This comparatively lower performance was observed since the dataset contained a limited number of stain samples, which are hard to obtain and create. Nevertheless, the precision, recall and f1-scores for all the classes are relatively high, reflecting the success of the model.

4 $$\begin{aligned}{} & {} Accuracy(A) = \frac{tp+tn}{tp+fp+tn+fn} \end{aligned}$$

5 $$\begin{aligned}{} & {} Precision(P) = \frac{tp}{tp+fp} \end{aligned}$$

6 $$\begin{aligned}{} & {} Recall(R) = \frac{tp}{tp+fn} \end{aligned}$$

7 $$\begin{aligned}{} & {} F_{measure}(F_1) = \frac{2 \times precision \times recall}{precision + recall} \end{aligned}$$

8 $$\begin{aligned}{} & {} mAP =\frac{1}{n} \sum _{i=1}^{n} AP_i \end{aligned}$$

9 $$\begin{aligned}{} & {} AP =\int _{0}^{1}d(r)\,\textrm{d}r. \end{aligned}$$

**Table 1:** Performance metrics of the proposed detection model.

Type	Precision	Recall	F1-score	mAP@0.5 (%)
All	0.943	0.895	0.918	94.6
Solid	0.969	0.949	0.959	97.7
Liquid	0.958	0.937	0.947	97.7
Stain	0.90	0.80	0.847	88.5

#### Comparison of performance with different detection models

In order to assess the efficiency of our proposed model, the mean average precision (*mAP*) at *IoU* = 0.5 of the YOLOv5 architecture has been compared with other popular object detection networks such as Faster R-CNN Resnet^39^ and Mobilenet-SSDv2^40^. Both networks have been trained using the same dataset for a similar amount of time, to conduct an unbiased evaluation.

Table 2 shows the statistical measures observed during the testing. *mAP*@0.5 clearly indicates that YOLOv5 outperforms the other two frameworks by a decent margin. The results obtained here also coincide with literature^41–43^ where YOLOv5 performs the best, followed by Faster R-CNN Resnet and MobilenetSSDv2. YOLOv5 has the lowest inferencing time^44,45^ compared to Faster R-CNN Resnet and MobilenetSSDv2. Hence, YOLOv5 shows the best performance in terms of *mAP* and inference time, making it the ideal architecture for the use case. Furthermore, YOLOv5 is optimized for low computationally powerful hardware, which makes it possible to deploy the YOLOv5 on a mobile robot for real-time dirt detection and classification.

**Table 2:** Performance comparison of different architectures.

Model	Precision	Recall	F1-score	mAP@0.5 (%)
YOLOv5 (proposed)	0.943	0.895	0.918	94.6
MobilenetSSDv2	0.626	0.60	0.613	63.0
Faster R-CNN ResNet	0.809	0.631	0.709	81.0

#### Validation of dirt tracking feature

**Figure 8:**
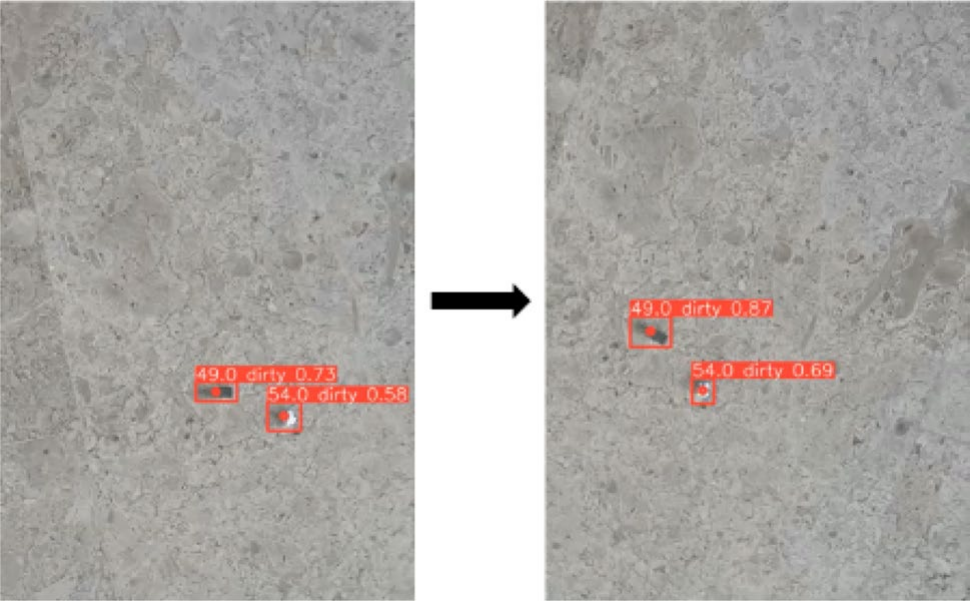
Tracked detections in sequential frames using real-time video footage. Here, the class ‘dirty’ on the bounding boxes refers to solid dirt.

For the entire proposed system to work, it is essential to detect the dirt in the first place and then track it using the output of the dirt detection module. Therefore, the main goal of the dirt tracking module is to track and count dirt objects with high speed and accuracy. The output of the dirt tracking module will be a dirt-bounding box with a sequence number. This testing was conducted by feeding a video to the system. From Fig. 8, it can be seen that the YOLOv5 + DeepSORT system is able to track and count the various dirt objects successfully. Furthermore, the proposed system can output the entry/exit time and allow robots to sync with the tracked detection for mapping purposes.

Overall, the proposed system works relatively fast with low inference time. According to the experiments, the inference time for the proposed model was found as 0.947 s where YOLOv5 and DeepSORT individually consumed 0.348 and 0.599 s, respectively. Hence, the proposed method is suitable for real-time dirt detection, classification and tracking for mapping the dirt distribution using a mobile inspection robot.

### Validation of dirt distribution mapping

**Figure 9:**
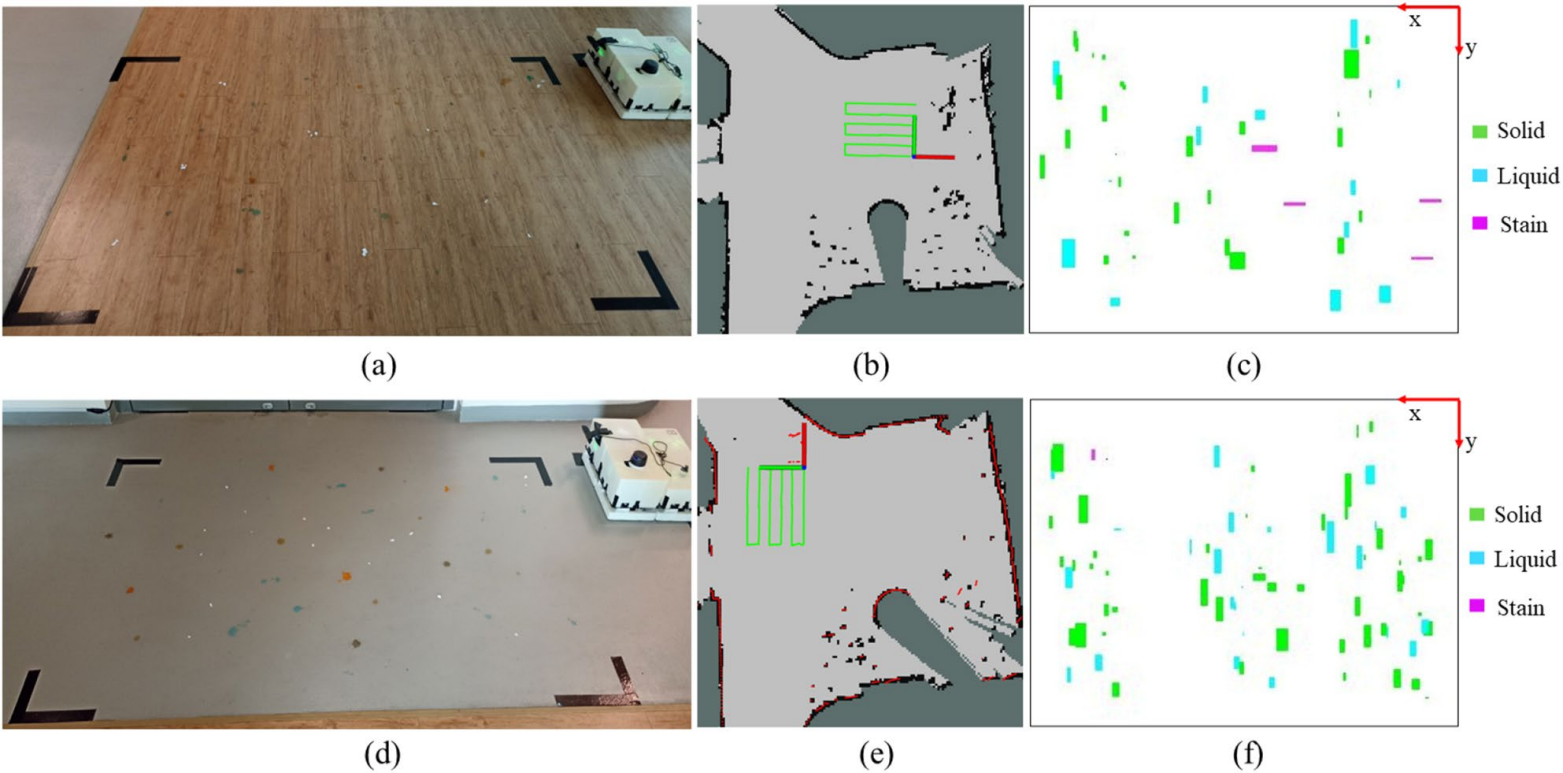
Two experiment cases to validate the dirt mapping ability: (**a**) environment used for the first case, (**b**) path robot has taken to cover the indented area in the first case, (**c**) dirt distribution map for first case, (**d**) environment used for the second case, (**e**) path robot has taken to cover the indented area in the second case, (**f**) dirt distribution map for second case.

In order to understand the real-time performance of the proposed system, the dirt distribution maps that indicates the type of dirt were generated for two selected test environments by navigating the inspection robot (Supplementary Video 1). The test environments consisted of two different floor patterns. Both environments had a rectangular shape with 175 $\times$ 250 cm dimensions. The first test environment is a wooden floor with a non-uniform pattern (See Fig. 9a). The second test environment is a uniform floor with grey colour but had a rough surface (See Fig. 9d). For the experiments, we have set the parameters such as lighting conditions, camera angle, camera location and robot’s speed, and the width and the length of the Zigzag path to ensure the complete and effective coverage of the selected area. These parameters were decided through a trial and error and kept constant for both experiments. Insights from this sensitivity analysis have enabled us to find out how to tune the performance of the proposed system. The traced navigation paths of the robot in the first and second environments are shown in Fig. 9b,e, respectively. The resultant dirt distribution maps are shown in Fig. 9c,f for the first and second environment, respectively.

The resultant dirt distribution maps were then compared with the actual distribution of the environment to evaluate the accuracy. The dirt map of the first environment where the floor had a non-uniform pattern has an accuracy of 76.7% while the accuracy of the map of the second environment (uniform floor) is 85.3%. The reason for this variation was that the contrast of the object made it more visible in the grey colour uniform floor. Even though the second environment had a uniform pattern, the surface was not smooth, inducing vibrations on the robot during navigation. As a result of this vibration, a comparatively larger detection time was observed in the second environment. Overall, the system had an accuracy of 81.0% in identifying and locating the dirt elements. It should be noted that, we could not compare the accuracy of dirt maps generated by our proposed approach with existing methods that can map dirt distribution into the three categories of solid, liquid, and stain, as no such methods currently exist in the literature to the best of our knowledge. Nevertheless, the results demonstrated the effectiveness and practicality of our proposed approach in accurately categorizing dirt and generating corresponding distribution maps that would be useful for a collaborative cleaning system with a set of heterogeneous robots.

On both floors, we also conducted experiments with different lighting conditions (i.e., natural and artificial lighting). In the events of artificial lighting, the detection became inaccurate, and objects were not even visible to the camera in some instances. This performance degradation was due to the shinning of the floor. Hence, the system is proposed to be used in diffused lighting situations for higher accuracy. During the experiments, we observed that some of the dirt were detected when they were far away and became undetected when they were closer. This behaviour was due to the variation of the dirt size perceived by the camera, where the size of dirt in the training data set influenced object detection. These limitations can be overcome by diversifying the training dataset with the dirt of different sizes and allowing the model to generalise better.

## Conclusion

Artificial intelligence and robotics play a major role in autonomous cleaning applications. Floor-cleaning robots with different cleaning functionalities have been introduced. A floor cleaning robot could fail or damage when cleaning incompatible dirt types. In order to overcome this issue, a vision-based dirt distribution mapping method for an inspection robot has been proposed in this paper.

The proposed method uses a detection and tracking scheme which consist of the YOLOv5 and DeepSORT algorithms. These models were trained to detect and classify dirt elements belonging to solid, liquid and stain classes and determine their positions. The developed method has been implemented in a mobile robot to evaluate dirt mapping accuracy. According to the experimental results, the developed method can create dirt distribution maps with sufficient accuracy. These dirt distribution maps would be useful for efficiently guiding collaborative robots with different cleaning functions. The scope of this work is limited to creating dirt distribution maps using a vision-enabled inspection robot. As future work, we expect to extend the system for the optimum path planning for guiding a set of cleaning robots based on these dirt distribution maps. The travelling salesman problem can be a potential approach for the optimum path planning based on the information provided by the dirt distribution maps.

### Supplementary Information

Supplementary Information 1.

Supplementary Video 1.

## Data Availability

The datasets generated during and/or analysed during the current study are available from the corresponding author on reasonable request.
